# Prognostic Value of 12-Leads Electrocardiogram at Emergency Department in Hospitalized Patients with Coronavirus Disease-19

**DOI:** 10.3390/jcm11092537

**Published:** 2022-04-30

**Authors:** Giulia Savelloni, Maria Chiara Gatto, Francesca Cancelli, Anna Barbetti, Francesco Cogliati Dezza, Cristiana Franchi, Martina Carnevalini, Gioacchino Galardo, Tommaso Bucci, Maria Alessandroni, Francesco Pugliese, Claudio Maria Mastroianni, Alessandra Oliva

**Affiliations:** 1 Department of Public Health and Infectious Diseases, Sapienza University of Rome, 00185 Rome, Italy; giu.savelloni@uniroma1.it (G.S.); francesca.cancelli@uniroma1.it (F.C.); anna.barbetti@uniroma1.it (A.B.); francesco.cogliatidezza@uniroma1.it (F.C.D.); cristiana.franchi@uniroma1.it (C.F.); martina.carnevalini@uniroma1.it (M.C.); claudio.mastroianni@uniroma1.it (C.M.M.); 2 Department of Clinical, Internal Medicine and Cardiovascular Sciences, Sapienza University of Rome, 00185 Rome, Italy; mariachiara.gatto@uniroma1.it; 3 National Institute for Infectious Diseases Lazzaro Spallanzani-IRCCS, 00149 Rome, Italy; 4 Department of General and Specialized Surgery “Paride Stefanini”, Sapienza University of Rome, 00161 Rome, Italy; gioacchino.galardo@uniroma1.it (G.G.); tommaso.bucci@uniroma1.it (T.B.); 5 Medical Emergency Unit, Sapienza University of Rome, Policlinico Umberto I, 00161 Rome, Italy; maria.alessandroni@uniroma1.it; 6 Anesthesiology and Intensive Care Group, Sapienza University of Rome, Policlinico Umberto I, 00161 Rome, Italy; francesco.pugliese@uniroma1.it

**Keywords:** COVID-19, electrocardiogram, SARS-CoV-2, electrocardiography, right ventricular strain, heart rhythm disorders, atrial fibrillation, emergency department

## Abstract

Background: Electrocardiogram (ECG) offers a valuable resource easily available in the emergency setting. Objective: Aim of the study was to describe ECG alterations on emergency department (ED) presentation or that developed during hospitalization in SARS-CoV-2-infected patients and their association with 28-day mortality. Methods: A retrospective, single-center study including hospitalized patients with SARS-CoV-2 was conducted. ECG was recorded on ED admission to determine: heart rhythm, rate, and cycle; atrio-ventricular and intra-ventricular conduction; right ventricular strain; and ventricular repolarization. A specialized cardiologist blinded for the outcomes performed all 12-lead ECG analyses and their interpretation. Results: 190 patients were included, with a total of 24 deaths (12.6%). Age (*p* < 0.0001) and comorbidity burden were significantly higher in non-survivors (*p* < 0.0001). Atrial fibrillation (AF) was more frequent in non-survivors (*p* < 0.0001), alongside a longer QTc interval (*p* = 0.0002), a lower Tp-e/QTc ratio (*p* = 0.0003), and right ventricular strain (*p* = 0.013). Remdesivir administration was associated with bradycardia development (*p* = 0.0005) but no increase in mortality rates. In a Cox regression model, AF (aHR 3.02 (95% CI 1.03–8.81); *p* = 0.042), QTc interval above 451 ms (aHR 3.24 (95% CI 1.09–9.62); *p* = 0.033), and right ventricular strain (aHR 2.94 (95% CI 1.01–8.55); *p* = 0.047) were associated with higher 28-day mortality risk. Conclusions: QTc interval > 451 ms, right ventricular strain, and AF are associated with higher mortality risk in SARS-CoV-2 hospitalized patients. ECG recording and its appropriate analysis offers a simple, quick, non-expensive, and validated approach in the emergency setting to guide COVID-19 patients’ stratification.

## 1. Introduction

Since the beginning of the SARS-CoV-2 global emergency in December 2019, more than 300 million cases and 5 million deaths have been recorded worldwide, and these numbers keep rising [1].

Multimorbidity, including past cardiovascular or pulmonary disease history and older age above all, have been previously associated with severity of infection and mortality [2]. On the other hand, the main respiratory features of COVID-19 come along with multiorgan complications, comprising cardiac injury, arrhythmias, and thromboembolism that worsen the outcome [3] in a vicious cycle fueled by the ongoing pro-inflammatory and hypoxic status and the autonomic impairment likely driven by the ACE2-angiotensin pathway and the sympathetic-vagal imbalance [4].

Electrocardiographic abnormalities have been observed in 99% of elderly and critically ill patients infected with SARS-CoV-2 [5]. These include a wide range of alterations spanning from arrhythmias, most frequently atrial fibrillation (AF) [6], to repolarization abnormalities, ST segment, and QT interval, among others, and to electrocardiographic signs of right ventricular overload and strain, such as S_1_Q_3_T_3_ sign or inferior leads T wave inversion, which reflects the associated lung involvement and is already linked to higher disease burden [7].

Despite the bulky amount of data, a comprehensive analysis of ECG parameters on emergency presentation in COVID-19 patients is missing, as either attention is focused on specific ECG abnormalities, or solid evidence on alterations is still lacking. ECG recording represents the first step of the cardiological assessment and can prove essential for patients’ risk stratification in the ongoing emergency frame, being a handy, inexpensive, and widely available tool.

Therefore, this study aims to describe the prevalence and type of electrocardiographic alterations at emergency department (E.D.) arrival in subsequently hospitalized SARS-CoV-2-infected patients and to investigate the possible association between ECG parameters and 28-day mortality after adjusting for variables, including age, sex, comorbidities, and laboratory findings that could influence the endpoint.

## 2. Materials and Methods

### 2.1. Study Design and Setting

A monocentric, retrospective study was conducted at Azienda Ospedaliero Universitaria Policlinico Umberto I, a tertiary care hospital with 1235 beds, the seat of “Sapienza” University of Rome Medical School, between March 2020 and January 2021.

### 2.2. Inclusion and Exclusion Criteria

**Figure 1:**
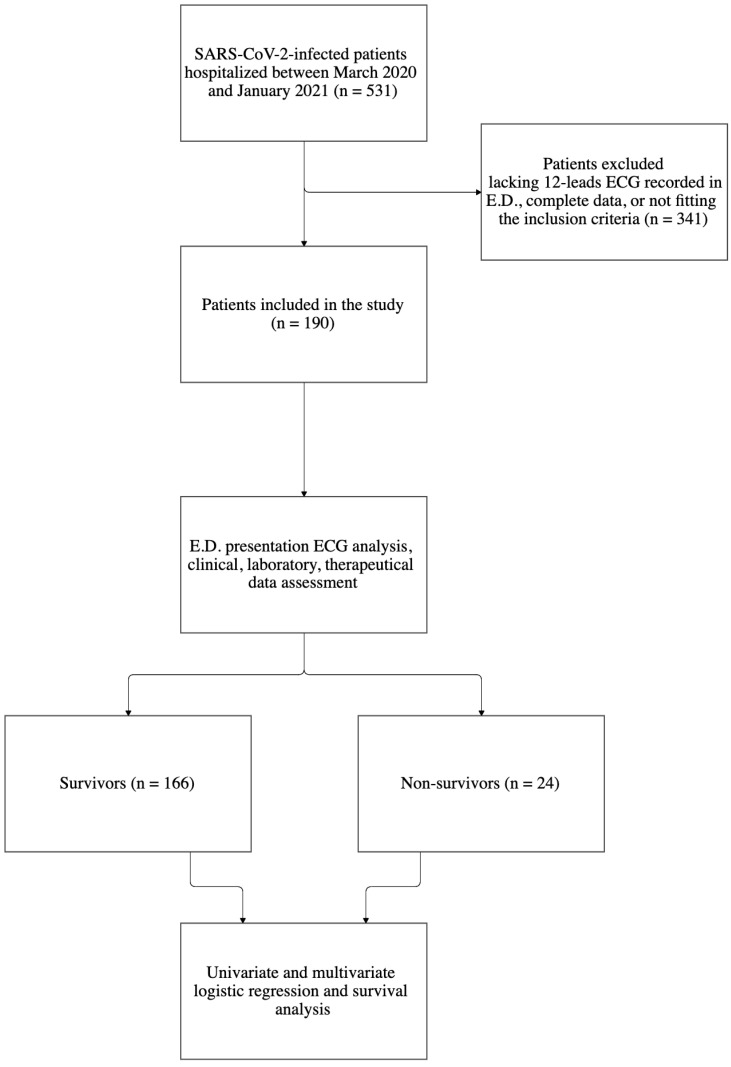
Flow-chart of patients’ recruitment and study course. ED, emergency department; ECG, electrocardiography.

Patients over 18 years of age with SARS-CoV-2 infection confirmed by rapid antigen or molecular (Real-Time PCR) nasopharyngeal swab test subsequently admitted from E.D. to Infectious Diseases COVID-19 hospital wards in the abovementioned period were initially included in the analysis, for a total of 531 patients. Underage or discharged patients, patients with no laboratory proven infection, or those admitted to wards other than infectious diseases ward (I.D.) (ICU, Intensive Care Unit; Pneumology, Internal Medicine ward) as well as patients receiving drugs potentially elongating the QT as well as >48 h of azithromycin or hydroxychloroquine were excluded. In addition, patients were excluded whether data were incomplete for study purpose or E.D. recorded standard twelve-lead ECGs were missing. Accordingly, the proposed criteria led to 341 excluded and 190 included patients (Figure 1).

### 2.3. Data Extraction and Definitions

Patients’ data were anonymously recorded from medical reports into an electronic spreadsheet for the following statistical analysis. These consisted of demographics; comorbidities included in the Charlson Comorbidity Index (CCI); plus systemic hypertension, AF, and asthma; vital signs recorded in E.D., including relative bradycardia (defined as copresence of body temperature ≥ 38.3 °C and heart rate (HR) < 90 bpm) [8,9], symptoms presentation and duration; laboratory tests performed on E.D. arrival, including PaO_2_/FiO_2_ ratio; potential ICU stay during hospitalization; in-hospital and 28-day mortality, length of hospital stay, and therapy administered against SARS-CoV-2. 

### 2.4. ECG Analysis

All 12-lead ECG analyses and their interpretation were performed by a specialized cardiologist (M.C.G.) who was blinded for the outcomes. The following parameters were retrieved: heart rhythm, heart rate (expressed as bpm), and heart cycle (RR interval and its standard deviation (RR SD), expressed as ms), atrio-ventricular and intra-ventricular conduction parameters ((PR interval, QRS length, both expressed as ms, presence of AV or IV blocks including left anterior hemi-block (LAH), left posterior hemi-block (LPH), right or left bundle branch block (RBBB or LBBB)), morphological evaluation with particular emphasis on right ventricular strain (S_1_Q_3_T_3_ or T_3_ alone pattern), and ventricular repolarization (QTc and Tp-e dispersion, Tp-e/QT, and Tp-e/QTc ratios).

For each subject, clinical parameters, including heart rate, pulse, and body temperature, were measured every 6 h. In case of heart rate and pulse alteration, a 12-lead ECG was recorded.

Cardiovascular events, including cerebrovascular and thromboembolic ones, and arrhythmias eventually experienced while hospitalized during the study period were additionally collected.

The study was conducted according to the principles of Declaration of Helsinki and was approved by the local Ethics Committee (ID Prot. 109/2020). The need for informed consent was waived since all data were retrospectively extracted.

### 2.5. Definitions

Cardiovascular events during hospitalization were defined as the onset of new ischemic/embolic events, such as pulmonary thrombo-embolism by lung CT scan, acute cerebral ischemia, acute limb ischemia, or the development of myocardial infarction, Takotsubo syndrome, myocarditis.

Heart rhythm disorders included the new onset of atrial fibrillation, supraventricular tachycardia, bradycardia, pairs of ventricular premature beats, and ventricular tachycardia.

Relative bradycardia was defined as heart rate < 90 bpm and concomitant fever (tympanic temperature ≥ 38.3 °C) [9].

Daytime bradycardia was defined as mean heart rate < 60 bpm recorded three times a day.

Right ventricular strain was defined as the presence of S_1_Q_3_T_3_ pattern (prominent S wave in lead I, Q wave in lead III, and negative T wave in lead III) or negative T wave alone in leads V1–V3 or II, III, or aVF with or without ST depression [7].

The QT interval was defined as the interval from the onset of the QRS complex to the end of the T wave and expressed as ms. Corrected QT interval (QTc) was calculated according to Bazett formula: QTc = QT√(RR interval) [10]. In the presence of intra-ventricular conduction disorder, the QTc interval was calculated as follows: QT − 155 × (60/heart rate − 1) − 0.93 × (QRS − 139) + k (where k was −22 ms for men and −34 ms for women) [11]. Prolonged QTc was defined as values > 440 ms and >460 ms in men and women, respectively.

QT dispersion, which reflects regional differences in myocardial refractoriness and predict cardiac dysrhythmias [12], was expressed as interlead QT interval differences within a 12-lead ECG and calculated as the difference between minimum and maximum QT interval.

Furthermore, regional differences in myocardial refractoriness were calculated also by means of Tp-e interval (expressed in ms and calculated from the peak of T wave to the end of T wave) [13]. Measurements of Tp-e interval were performed from precordial leads, and the longest Tp-e interval was recorded. Tp-e dispersion was defined as the difference between the maximum and minimum Tp-e interval in the precordial leads (V1–V6) during a single beat [13]. From the abovementioned parameters, we obtained Tp-e/QT and Tp-e/QTc ratios [13].

### 2.6. Statistical Analysis

Statistical analysis was performed using STATA^®^ software, v. 15 (StataCorp); charts were generated using Microsoft Office^®^ and Graphpad Prism^®^. Continuous data are expressed as median and interquartile range (IQR) values and categorical variables as numbers and percentage values. Categorical variables were compared using χ^2^-test or Fisher’s exact test and continuous variables using Student’s *t*-test or Mann–Whitney U test as appropriate.

In the subgroup of patients with troponin levels available, Spearman correlation analyses between troponin levels and PaO_2_/FiO_2_, D-dimer, CRP, and lymphocytes count, expression of respiratory failure and inflammation during COVID-19, respectively, were performed. According to the reference values available at the laboratory of our hospital, abnormal levels of troponin corresponded to levels > 0.014 μg/L.

Multivariate Cox regression models were used to determine the hazard ratios (HR) for mortality within 28 days from admission of the included variables accounting for covariables. Statistically and clinically relevant variables on univariate analysis were evaluated for the determination of the final multivariate model. To find the optimal cut-off of the QTc value associated with the highest sensitivity and specificity in the prediction of 28-day outcome, the Youden’s Index was used. The resulting value was further inserted in the final model. Twenty-eight-day survival curves were plotted using the Kaplan–Meier method and compared using the *log*rank test. For all the statistical analyses, *p* < 0.05 was considered significant.

## 3. Results

### 3.1. Demographics and Outcome

Overall, 190 patients were included in the study, with 83/190 (44%) females (Table 1). Median age was 66 (IQR 55–80) years. Twenty-four deaths were recorded, accounting for 12.6% global mortality rate, with 87.5% (21/24) death rate recorded within 28 days from admission. Median length of hospitalization was 18 days (IQR 11–28). As for deceased patients’ subgroup, age resulted significantly higher when compared to survivors (median (IQR), 83.5 (79–88]) vs. 64 (54–77) years; *p* < 0.0001), while no significant difference was observed regarding sex. No deaths were recorded within 48 h from E.D. arrival; therefore, this variable was not included in the study. Patients hospitalized during the first pandemic wave in Italy (February–June 2020) were evenly distributed among the two compared subgroups (89/166 (53.6%) vs. 10/24 (41.7%); *p* = 0.273). This reduces possible bias in different diagnostic and therapeutic management of SARS-CoV-2 infection over time.

### 3.2. Baseline Comorbidities

Systemic hypertension arose as the most frequent comorbidity overall (109/190 (57.3%) patients). Calculated CCI was significantly higher in non-survivors (median (IQR), 8 (8–10)) when compared to survivors (3 (1–6); *p* < 0.0001). Besides hypertension, myocardial infarction (MI) was the most common cardiovascular comorbidity (27/190 patients, 14.2%), followed by AF (20/190 patients, 10.5%) and chronic heart failure (CHF) (12/190 patients, 6.3%), which was more prevalent in the deceased subgroup, where AF was recorded in 7/24 patients (29.1% vs. 7.8%; *p* < 0.0001), MI in 6 (25% vs. 13%; *p* = 0.084), and CHF in 3 (12.5% vs. 5.4%; *p* = 0.160) (Table 1).

### 3.3. Clinical Presentation and Characteristics at E.D.

Fever was the most prevalent symptom in both the overall population (149/190 patients (78.4%)) and among survivors (136/166 (82%) vs. 13/24 (54.2%) patients; *p* = 0.002), whereas dyspnea was the most prevalent among deceased although not statistically significant (16/24 (66.7%) vs. 85/166 (51.2%) patients; *p* = 0.164). Moreover, in the latter subgroup, median time between symptoms onset and E.D. presentation was significantly shorter than in survivors (median (IQR), 1.8 (0–5.5) vs. 6 (2–9) days; *p* = 0.039). Relative bradycardia was registered in 22/190 patients (11.6%) and, though more frequent among survivors (21/166 patients (12.6%)), resulted as not statistically relevant (*p* = 0.225).

Turning to laboratory tests, PaO_2_/FiO_2_ ratio on triage arterial blood gas was lower in non-survivors (median (IQR), 302 (243–367) vs. 357 (314–424); *p* = 0.0007), together with high white blood cells count (7180 (5070–8820) vs. 5755 (4502–7790) cells/µL; *p* = 0.009) and neutrophils count (5465 (3647–7662) vs. 4150 (3110–5995) cells/µL; *p* = 0.0038), anemia (median Hb (IQR) 11.3 (10.2–14.5) vs. 13.8 (12.7–14.9) g/dL; *p* = 0.0004), high D-dimer (1995 (1012–3198) vs. 777 (429–1469) U/L; *p* = 0.0012) and LDH (330 (268–447) vs. 275 (213–349) U/L; *p* = 0.0001), and low serum albumin (32 (30–35) vs. 38 (35–41) g/L; *p* < 0.0001). Regarding serum troponin T, the test was available on admission in 125/190 patients only and was higher in non-survivors (0.031 (0.021–0.04) vs. 0.012 (0.007–0.0245) μg/L; *p* = 0.084). Table 1 resumes clinical characteristics and laboratory tests of the included patients.

### 3.4. ECG Findings on E.D. Admission

Table 2 shows the ECG findings registered on arrival recordings. Even though sinus rhythm was the most frequently recorded rhythm in both subgroups, supraventricular arrhythmias were more prevalent in non-survivors, as AF was observed in 33.3% of patients (8/24 (33.3%) vs. 10/166 (6%); *p* < 0.0001) and paroxysmal supraventricular complexes (PSVC) in 20.8% (5/24 (20.8%) vs. 9/166 (5.4%) patients; *p* = 0.0193). Mean RR interval was reduced, albeit not significantly, in non-survivors (median (IQR), 637.5 (570–762) vs. 767 (664–875) ms; *p* = 0.006) as well as its standard deviation (18 (11–30) vs. 20 (13.4–35) ms; *p* = 0.414). Regarding ventricular conduction parameters, the only significant difference between the subgroups was observed for the higher prevalence of left anterior hemiblock (9/24 (37.5%) vs. 28 (19.8%) patients; *p* = 0.0258) in non-survivors, who also recorded a higher occurrence of right ventricular strain as S_1_Q_3_T_3_ pattern (7/24 (29.1%) vs. 18/166 (10.8%) patients; *p* = 0.013) or as single-components inverted T wave in DIII (T_3_) (15/24 (62.5%) vs. 45/166 (27.1%) patients; *p* < 0.0001) and prominent S wave in DI (S_1_) (9/24 (37.5%) vs. 30/166 (18%) patients; *p* = 0.034). Median QTc interval duration was longer in non-survivors (436.8 (435–487) vs. 428 (402–447) ms; *p* = 0.0002), resulting in lower Tp-e/QTc ratio (0.2 (0.158–0.198) vs. 0.22 (0.211–0.233); *p* = 0.0003). Prolonged QTc was observed in 55 (28.9%) subjects, higher in non-survivors than survivors (39 (23.49%) vs. 16 (66.6%), *p* < 0.0001). Following Youden’s index, the optimal cut-off of QTc differentiating 28-day survivors from non-survivors was 451 ms (sensitivity 61.9%, specificity 79.2%, AUROC 0.70 (0.59–0.81)).

### 3.5. Arrhythmic Events during Hospitalization

Table 3 displays cardiovascular events developed during hospitalization and comparison between survivors and deceased. Cumulative AF, comprising that registered both on E.D. recording and acquired as inpatients, was significantly prevalent in non-survivors (11/24 (45.8%) vs. 18/166 (10.8%) patients; *p* < 0.0001) as new onset in-hospital AF although not relevant (3/24 (12.5%) vs. 8/166 (4.8%) patients; *p* = 0.1477). On the other hand, daytime bradycardia was more frequent in survivors (2/24 (8.3%) vs. 28/166 (16.9%); *p* = 0.284).

### 3.6. Anti-SARS-CoV-2 Therapies

In the non-survivor subgroup, a higher rate of patients was treated with therapeutic dose LMWH (100 U/kg/12 h s.q.) (10/24 (41.7%) vs. 33/166 (19.9%) patients; *p* = 0.0045) and with systemic corticosteroids (17/24 (70.8%) vs. 72/166 (43.4%), *p* = 0.045), while administration of macrolides, HCQ, and prophylactic dose LMWH (4.000 UI/24 h s.q.) was uniform within the two subgroups, as shown in Table 1. As for remdesivir, the study sample (35/190 patients overall (18.4%) and 1/24 non survivors (4.1%)) does not allow a coherent analysis among groups although remdesivir was associated with bradycardia development with no link to mortality.

### 3.7. Troponin Levels and Correlation Analyses

Troponin levels at hospital admission was available in 125/190 (65.8%) subjects. Abnormal levels of troponin (>0.014 μg/L) were associated with AF and QTc (*p* = 0.021 and 0.036, respectively) but not with right ventricular strain (*p* = 0.94) and tended to be associated with abnormalities at admission ECG (*p* = 0.08).

**Figure 2:**
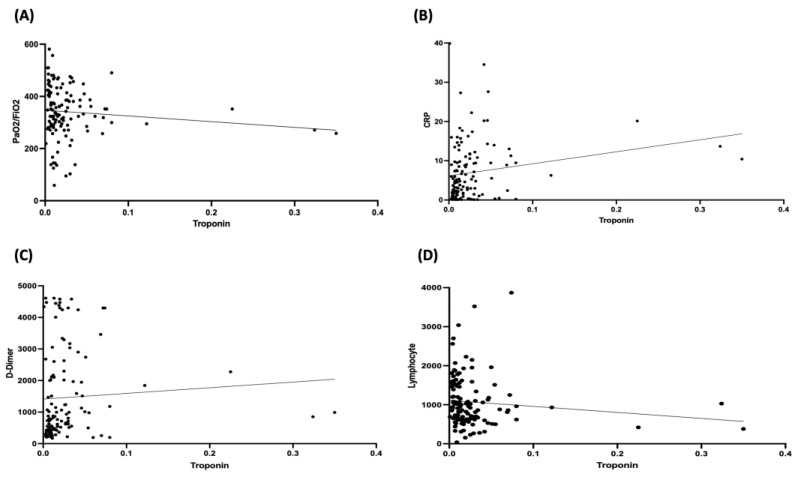
Correlation analyses between troponin values and PaO_2_/FiO_2_ (Panel **A**), CRP (Panel **B**), D-dimer (Panel **C**), and lymphocyte count (Panel **D**) at hospital admission. CRP, C-reactive protein (expressed as mg/dL). D-dimer, troponin, and lymphocyte count are expressed as ng/mL, μg/L, and lymphocytes/μL, respectively.

A positive correlation between troponin levels and D-dimer and CRP at hospital admission was found (r = 0.34, *p* = 0.0001 and r = 0.31, *p* = 0.0006, respectively), whereas a negative correlation was observed with PaO_2_/FiO_2_ (r = −0.18, *p* = 0.045) and lymphocyte count (r = −0.27, *p* = 0.023) (Figure 2).

### 3.8. Kaplan–Meier Survival Curves for 28-Day Mortality

**Figure 3:**
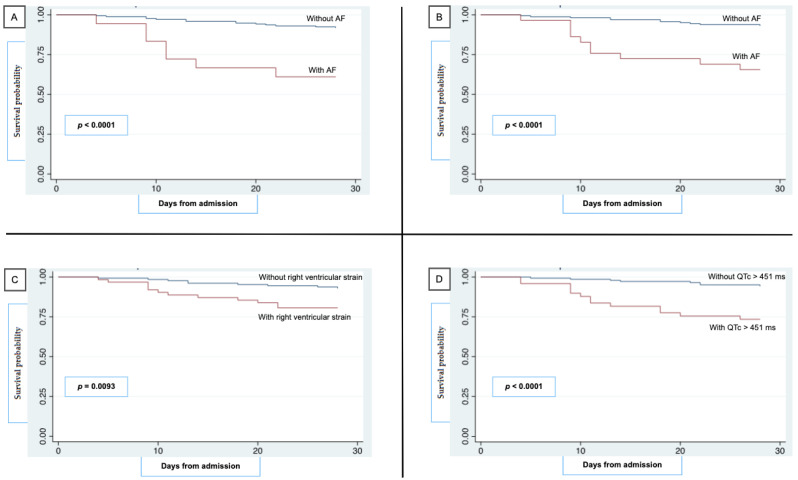
Estimated 28-day survival probability after hospital admission for patients with and without AF rhythm detection on ED presentation (**A**), on ED presentation and with in-hospital onset (**B**), right ventricular strain (**C**), and prolonged QTc interval (**D**). ED, emergency department; AF, atrial fibrillation.

Figure 3 depicts survival curves for 28-day mortality and *log*rank test analysis with *p*-values. Among electrocardiographic findings, AF on E.D. admission recording (*p* < 0.0001) or developed during hospitalization (*p* = 0.0409) or considered together as cumulative AF (*p* < 0.0001) were associated with lower 28-day survival rates for right heart strain (*p* = 0.0093) and QTc value > 451 ms (*p* < 0.0001). Relative bradycardia was not significantly different between survivors and deceased (*p* = 0.3148). In addition, age > 65 y (*p* = 0.0002), CRP over 4 mg/dL (*p* = 0.0023), D-dimer over 850 U/L (*p* = 0.0035), and serum albumin below 35 g/L (*p* < 0.0001) on E.D. admission laboratory tests were associated with lower 28-day survival rates.

### 3.9. Multivariate Adjusted Cox Hazard Regression Model of Independent Factors Associated with 28-Day Mortality

Statistically (*p* < 0.05) and clinically relevant variables on univariate analysis were evaluated for the determination of hazard ratios (HRs) for 28-day mortality. Following Youden’s index results, QTc value > 451 ms was considered in the final model. AF detection on E.D. arrival ECG or its in-hospital development (HR 3.02 (95% CI 1.03–8.81); *p* = 0.042), QTc > 451 ms (HR 3.24 995% CI 1.09–9.62); *p* = 0.033), and right ventricular strain (HR 2.94 (95% CI 1.01–8.55); *p* = 0.047) were associated with higher 28-day mortality risk after adjustment for age, sex, cardiac and pulmonary comorbidities (MI, systemic hypertension, CHF, COPD), and laboratory tests (*p*/F ratio, D-dimer) that proved clinically pertinent and could potentially influence the outcome (Table 4).

Additional multivariable analysis performed on the subgroup of patients with troponin levels availability showed that abnormal levels of troponin at hospital admission were not associated with 28-day mortality.

## 4. Discussion

This study supports the association between AF, QTc, and right ventricular strain and higher mortality risk in patients hospitalized with SARS-CoV-2 infection. Two years after the beginning of the COVID-19 pandemic, the international community still faces a rise in both global cases and death toll. Therefore, the detection of these ECG findings aims to provide clinicians with an additional tool for patients’ stratification in this emergency framework. Our study shows a 12.6% mortality rate, higher than the average reported in Italy [14] but lower than the 20% rate reported among people aged over 80 years by the Italian National Health Institute [15]. This could reflect the presence of patients hospitalized during the first pandemic wave in our study, when mortality rates were considerably higher in our country, dropping from 59.1 deaths/100,000 inhabitants to 3.1/100,000 after June 2020 [16]. Moreover, median age in non-survivors was noticeably high (83.5 years). This contributes to the high age-adjusted CCI, matching a 0% 10-year survival rate for the median CCI registered [17]. In addition, age confirms its largely described association with 28-day mortality, being the single risk factor mostly associated with disease severity and mortality in SARS-CoV-2 infection [2]. Male sex was instead not significantly associated with mortality in our study, as previously reported [2]. Systemic hypertension was recorded as the most frequent comorbidity in our study but with higher rates than previously reported in systematic review analysis and with no link to higher mortality risk [18].

Even though COVID-19 is mostly considered a respiratory disease, heart rhythm alterations are recognized as risk factors for mortality. Arrhythmias including AF have been associated with a 3.1-fold higher mortality risk and 29% risk rate for severe disease in SARS-CoV-2-infected patients [19], and a recent meta-analysis including 187.716 COVID-19 patients confirmed that AF was associated with a 4-fold higher risk of death [20]. AF prevalence in non-survivors was 45.8%, accounting for both AF rhythm on E.D. presentation and in-hospital development, higher than the rates reported by the Italian National Health Institute (24.5%) for COVID-19 patients [15]. However, the median age in this subgroup was remarkably high, and AF incidence increases with age [21].

Not only AF rhythm but also QTc > 451 ms proved to be independently associated with 28-day mortality after adjustment for covariables. The latter one has been under special surveillance since the beginning of the pandemic because it is a well-known adverse event of HCQ and azithromycin, which was previously administered against SARS-CoV-2 [22]. Regardless, the ECGs included in our study were recorded on E.D. admission, prior to drug administration.

Furthermore, hypoxic stress and lung damage, its related pulmonary hypertension and right ventricular heart strain, in addition to the high rates of pulmonary thromboembolism (PTE) [4] registered in COVID-19 patients are revealed by McGinn-White sign (S_1_Q_3_T_3_) or inverted T wave on ECG recordings. This has already been described as a negative prognostic factor in patients with non-COVID19-related PTE [23] and in our study was independently associated with 28-day mortality. The T_3_ sign alone, indeed, was included in the right ventricular strain analysis as a strain mark and a recognized negative prognostic factor [24]. Our findings match those described by Elias et al. [25], who linked the presence of AF and right ventricular strain on ECG recorded on E.D. admission to higher mortality risk and need for mechanical ventilation, with high prognostic value when paired with spO_2_ ≤ 95% and RR > 20 bpm. Concerning laboratory tests on E.D. admission, low PaO_2_/Fio_2_ ratio, anemia, low serum albumin, leukocytosis with neutrophilia, elevated LDH, D-dimer, and CRP were prevalent in non-survivors, as previously described [26]. Though with no statistically relevant difference between the subgroups, we observed lymphopenia (median 895 cells/µL) as well [27]. As for survival curves, a drop of survival rates is observed around the 10th day of hospital stay not only for high right ventricular strain, AF, and QTc interval prolongation (Figure 3) but also for serum albumin < 35 g/L, D-dimer > 850 U/L, and CRP > 4 mg/dL. At the same time, median time from symptoms onset to E.D. presentation in non-survivors was 1.8 days. This goes along with the natural history of SARS-CoV-2 infection that pinpoints days 10–14 as the most susceptible for a potential shifting toward a worse outcome, as the immune response triggers and carries out alveolar and systemic endothelial damage [28].

A total of 15.8% of patients developed sinus bradycardia during hospitalization. This was not linked to higher mortality rates, as previously described [29], even though it confirmed its association with remdesivir administration, recently proposed as protective against COVID-19 [30] and already labelled as transient and self-limiting [31]. Moreover, the high rates of LMWH administration (91.7%) in non-survivors as well as systemic steroids (70.8%) could imply the disease severity underneath, as already described for elevated CCI and AF rhythm rates with its drug burden. As mentioned, a subgroup of the included patients was hospitalized during the so-called “first pandemic wave” with different management strategies. This does not limit our study, as mortality rates are uniform in time, and our primary endpoint was ECG analysis and not the therapeutic options.

Autonomic dysfunction is shown throughout the lower heart rate variability registered in non-survivors as RR interval, which reflects the sympathovagal balance interacting with IL-6 and the ongoing pro-inflammatory boost [32,33]. This has shown predictive value for CRP elevation during SARS-CoV-2 hospitalization [34] and persistency in post-COVID syndrome [35].

Statistically significant correlations were found between troponin values and specific parameters of COVID-19, such as PaO_2_/FiO_2_ (expression of respiratory failure), D-dimer, CRP, and lymphocytes (expression of inflammation during infection). Furthermore, abnormal troponin levels were associated with AF and QTc and, overall, tended to be associated with abnormal ECG on hospital admission. Taken together, these findings suggest that the possible myocardial damage, expressed by the troponin values, may be directly related to the infection itself rather than to a previous cardiac problem. Nevertheless, although troponin represents a marker of myocardial injury, it may be unreliable when considered alone, and therefore, it should be analyzed only when combined with additional clinical and laboratory parameters.

This study shows several limitations. Primarily, its retrospective design does not allow a confident generalizability. Despite controlling for demographic and clinical data, there could be other confounding variables not included in multivariate analysis, and the omission of radiologic parameters does not grant a strict clinical assessment of right heart strain and conceivable underlying pulmonary thromboembolism. Moreover, the temporal nexus between ECG findings and their development is missing, as we only analyzed the E.D. ECG, and certain abnormalities could have developed prior to SARS-CoV-2 infection; nevertheless, having these ECG abnormalities was associated with a worse prognosis. The potential association between ECG findings and serum troponin T levels could not be assessed, because of the few available TnT, as a former non-routine test on E.D. admission; nevertheless, only a statistical trend over significance was observed, and therefore, this variable was further excluded from the final model.

Lastly, in consideration of the test duration and emergency situation experienced by our country during the study period, we did not have the possibility to routinely perform cardiac MRI to identify myocardial alterations in hospitalized patients and to comprehensively collect the frequency of post-COVID-19 cardiovascular sequelae. Nevertheless, the aim of the present study was to investigate the electrocardiographic features on hospital admission and in the emergency setting and not the cardiovascular sequelae, which, in our opinion, deserve per se additional investigations.

To conclude, our study was conducted in an emergency, namely the first and second pandemic waves in Italy, prior to the development and approval of the anti-SARS-CoV-2 management strategies currently used. This supports the goal of investigate the untainted interaction of SARS-CoV-2 with heart rate and rhythm and, therefore, ECG, avoiding additional confounders such as new antiviral drugs and prophylactic treatments or the role of virus variants.

## 5. Conclusions

This study demonstrates the association between older age, AF, QTc, and right ventricular strain recorded on E.D. admission ECG and higher mortality risk after adjusting for cardiopulmonary comorbidities and disease severity markers. These results endorse the role of the ongoing cardiovascular alterations in SARS-CoV-2 infection, likely related to the direct and indirect action of virus and cytokine storm on cardiomyocytes and unbiased by therapeutic strategies. ECG recording and its appropriate analysis offers a simple, quick, non-expensive, and validated approach in the emergency setting to guide COVID-19 patients’ stratification in the ongoing pandemic frame, assisted by clinical and laboratory assessment.

## Figures and Tables

**Table 1:** General characteristics of study population.

Characteristics	Overall Population *n* (%) = 190 (100)	Survivors *n* (%) = 166 (87.4)	Non-Survivors *n* (%) = 24 (12.6)	*p*-Value
Demographics and outcome measures				
Age—median (IQR), years	66 (55–80)	64 (54–77)	83.5 (79–88)	<0.0001
Females—*n* (%)	83 (44)	72 (43)	11 (46)	0.820
First wave—*n* (%)	99 (52.1)	89 (53.6)	10 (41.7)	0.273
ICU stay—*n* (%)	12 (6.4)	10 (6)	2 (8.3)	0.676
Length of stay—median (IQR), days	18 (11–28)	19 (12–28)	13 (9–22)	0.160
Global deaths—*n* (%)	24 (12.6)	N.A.	24 (100)	N.A.
Within 48 h—*n* (%)	0 (0)		0 (0)	
Within 7 days—*n* (%)	4 (2.1)		4 (16.7)	
Within 14 days—*n* (%)	14 (7.4)		14 (58.3)	
Within 28 days—*n* (%)	21 (11)		21 (87.5)	
>28 days	3 (1.7)		3 (12.5)	
Comorbidities				
CCI—median (IQR)	4 (2–7)	3 (1–6)	8 (8–10)	<0.0001
Stage III–IV CKD—*n* (%)	60 (31.6)	49 (29.5)	11 (45.8)	0.156
Diabetes mellitus (I and II)—*n* (%)	38 (20)	35 (21)	3 (12.5)	0.412
Periph. vascular disease—*n* (%)	34 (18)	25 (15)	9 (37.5)	0.005
COPD—*n* (%)	28 (14.9)	23 (13.8)	5 (20.8)	0.325
Myocardial infarction—*n* (%)	27 (14.2)	21 (13)	6 (25)	0.084
Dementia—*n* (%)	27 (14.3)	11 (6.6)	16 (66.7)	<0.0001
Solid tumor—*n* (%)	21 (11)	14 (8.4)	7 (29.2)	0.015
CVA/TIA—*n* (%)	18 (9.5)	9 (5.4)	9 (37.5)	<0.0001
Chronic heart failure—*n* (%)	12 (6.3)	9 (5.4)	3 (12.5)	0.160
Hemiplegia—*n* (%)	8 (4.2)	3 (1.8)	5 (20.8)	<0.0001
Liver disease—*n* (%)	7 (3.7)	3 (1.8)	4 (16.6)	<0.0001
Hematologic malignancies—*n* (%)	5 (2.6)	1 (0.5)	4 (16.7)	0.001
AIDS—*n* (%)				
Systemic hypertension—*n* (%)	109 (57.3)	95 (57.2)	14 (58.3)	0.741
Atrial fibrillation *—*n* (%)	20 (10.5)	13 (7.8)	7 (29.1)	<0.0001
Asthma—*n* (%)	10 (5.3)	8 (4.8)	2 (8.3)	0.436
Clinical and laboratory findings at E.D.				
Days from symptoms onset—median (IQR), days	5.9 (2–9)	6 (2–9)	1.8 (0–5.5)	0.039
Vital signs				
BT—median (IQR), °C	37 (36.2–37.95)	37 (36.5–38)	36.5 (36–37.45)	0.252
SpO2—median (IQR), %	96 (94–98)	96 (94–98)	96 (93–97)	0.356
HR—median (IQR), bpm	87 (80–100)	86.5 (80–98)	90 (79–110)	0.191
PaO_2_/FiO_2_—median (IQR), *n*	352 (295–419)	357 (314–424)	302 (243–367)	0.0007
Relative bradycardia **—*n* (%)	22 (11.6)	21 (12.6)	1 (4.1)	0.225
Reported symptoms	37 (36.2–37.95)	37 (36.5–38)	36.5 (36–37.45)	0.252
Fever—*n* (%)	149 (78.4)	136 (82)	13 (54.2)	0.002
Dyspnea—*n* (%)	101 (53.1)	85 (51.2)	16 (66.7)	0.164
Cough—*n* (%)	89 (46.8)	84 (50.6)	5 (20.8)	0.009
Weakness/osteoarticular—*n* (%)	37 (19.6)	29 (17.5)	8 (33.3)	0.069
Gastrointestinal—*n* (%)	25 (13.2)	23 (13.8)	2 (8.3)	0.449
Anosmia/dysgeusia—*n* (%)	17 (8,9)	16 (9.6)	1 (4.1)	0.380
Laboratory tests				
Hb—median (IQR), g/dL	13.8 (12.4–14.9)	13.8 (12.7–14.9)	11.3 (10.2–14.5)	0.0004
WBC—median (IQR), /μL	5835 (4625–8220)	5755 (4502–7790)	7180 (5070–8820)	0.009
Neutrophils—median (IQR), /μL	4310 (3160–6335)	4150 (3110–5995)	5465 (3647–7662)	0.0038
Lymphocytes—median (IQR), /μL	895 (635–1285)	920 (650–1367)	795 (527–1120)	0.130
Serum albumin—median (IQR), g/L	37 (34–40)	38 (35–41)	32 (30–35)	<0.0001
LDH—median (IQR), UI/L	280 (217–356)	275 (213–349)	330 (268–447)	0.0001
Serum TnT–median (IQR), μg/L ^§^	0.014 (0.007–0.028)	0.012 (0.007–0.024)	0.031 (0.021–0.04)	0.084
D-dimer—median (IQR), ng/mL	822 (449–1947)	777 (429–1469)	1995 (1012–3198)	0.0012
CRP—median (IQR), mg/dL				
Serum creatinine—median (IQR), mg/dL	3.93 (1.1–9.46)	3.51 (0.95–9)	7.57 (3.81–12.64)	0.669
Anti-SARS-CoV-2 therapies				
Remdesivir—*n* (%)	35 (18.4)	34 (20.5)	1 (4.1)	0.054
Therapeutic dose LMWH—*n* (%)	43 (22.6)	33 (19.9)	10 (41.7)	0.0045
VTE prophylaxis LMWH—*n* (%)	92 (48.4)	80 (48.2)	12 (50)	0.132
Systemic steroids—*n* (%)	89 (47.6)	72 (43.4)	17 (70.8)	0.045
Anti-IL6—*n* (%)	36 (19.2)	29 (17.5)	7 (29.2)	0.217
Anti-JAK—*n* (%)	1 (0.5)	1 (0.6)	0 (0)	N.A.
PI—*n* (%)	32 (16.8)	30 (18)	2 (8.3)	0.229
Macrolides—*n* (%)	105 (55.3)	92 (55.4)	13 (54.2)	0.537
HCQ—*n* (%)	82 (43.4)	74 (44.6)	8 (33.3)	0.288

Abbreviations: ICU, intensive care unit; CCI, Charlson Comorbidity Index; CKD, chronic kidney disease; COPD, chronic obstructive pulmonary disease; CVA/TIA, cerebrovascular accident/transient ischemic attack; BT, body temperature; HR, heart rate; WBC, white blood cells; TnT, troponin T; CRP, C-reactive protein; LMWH, low molecular weight heparin; VTE, venous thromboembolism; PI, protease inhibitor; HCQ, hydroxychloroquine; IQR, interquartile range; N.A., not applicable; AIDS, Acquired Immuno Deficiency Syndrome; E.D., Emergency Department; LDH, lactate dehydrogenase; JAK, Janus kinase. * includes both permanent and paroxysmal atrial fibrillation; ** relative bradycardia at admission was defined as BT ≥ 38.3 °C and HR < 90 bpm. ^§^ available for 125/190 patients.

**Table 2:** Electrocardiography features at Emergency Department admission.

ECG Feature	Recordings *n* (%) = 190 (100)	Survivors *n* (%) = 166 (87.4)	Non-Survivors *n* (%) = 24 (12.6)	*p*-Value
Heart rhythm				
Sinus rhythm—*n* (%)	172 (90.5)	156 (94)	16 (66.7)	<0.0001
Atrial fibrillation—*n* (%)	18 (10)	10 (6)	8 (33.3)	<0.0001
Other arrhythmias *—*n* (%)	18 (9.5)	13 (7.8)	5 (20.8)	0.057
Heart rate and cycle				
RR interval—median (IQR), ms	752.5 (637.5–840)	767 (664–875)	637.5 (570–762)	0.006
RR interval SD—median (IQR), ms	21.2 (14.1–35.3)	20 (13.4–35)	18 (11–30)	0.414
HR—median (IQR), bpm	79.7 (71.4–94.1)	81 (73–90)	94 (79–105)	0.015
Ventricular conduction				
AVB—*n* (%)	20 (10.6)	15 (9)	5 (20.8)	0.081
QRS duration—median (IQR), ms	90 (84–102)	89 (83–101)	95 (90–110)	0.119
LAH—*n* (%)	42 (22.2)	28 (19.8)	9 (37.5)	0.0258
LPH—*n* (%)	2 (1)	2 (1.2)	0 (0)	1
RBBB—*n* (%)	22 (11.6)	18 (10.8)	4 (16.7)	0.407
LBBB—*n* (%)	6 (3.2)	4 (2.4)	2 (8.3)	0.122
Right ventricular strain				
S_1_Q_3_T_3_ sign—*n* (%)	25 (13.2)	18 (10.8)	7 (29.1)	0.013
S_1—_*n* (%)	39 (20)	30 (18)	9 (37.5)	0.034
Q_3_—*n* (%)	39 (20)	33 (19.9)	6 (25)	0.562
T_3_—*n* (%)	60 (31.6)	45 (27.1)	15 (62.5)	<0.0001
Ventricular repolarization				
QTc duration—median (IQR), ms	432.5 (412.2–452)	428 (402–447)	436.8 (435–487)	0.0002
QT maximum value, median (IQR), ms	390 (320–480)	390 (360–410)	380 (357.5–412.5)	0.987
QT minimum value, median (IQR), ms	370 (340–400)	378 (350–396.5)	360 (337.5–400)	0.776
QT dispersion, median (IQR), ms	10 (10–20)	10 (10–20)	10 (10–20)	0.397
Prolonged QTc **—*n* (%)	55 (28.9)	39 (23.49)	16 (66.6)	<0.0001
Tp-e dispersion—median (IQR), ms	20 (20–30)	19 (19–27)	20 (20–30)	0.458
Tp-e/QT—median (IQR)	0.234 (0.214–0.253)	0.236 (0.212–0.256)	0.2 (0.209–0.238)	0.126
Tp-e/QTc—median (IQR)	0.206 (0.182–0.218)	0.22 (0.211–0.223)	0.2 (0.158–0.198)	0.0003

Abbreviations: HR, heart rate; AVB, atrioventricular block; LAH, left anterior hemiblock; LPH, left posterior hemiblock; RBBB, right bundle branch block; LBBB, left bundle branch block; QTc, corrected QT interval; Tp-e, T wave peak-end; SD, Standard Deviation. * includes premature ventricular and supraventricular complexes and sinus tachycardia. ** prolonged QTc was defined as values > 440 ms and >460 ms in men and women, respectively.

**Table 3:** Cardiovascular events registered during hospitalization.

Cardiovascular Events Registered during Hospitalization	Overall Population *n* (%) = 190 (100)	Survivors *n* (%) = 166 (87.4)	Non-Survivors *n* (%) = 24 (12.6)	*p*-Value
Daytime bradycardia—*n* (%)	30 (15.8)	28 (16.9)	2 (8.3)	0.284
AF—*n* (%)	11 (5.7)	8 (4.8)	3 (12.5)	0.1477
Overall AF (E.D. + in-hospital)—*n* (%)	29 (15.3)	18 (10.8)	11 (45.8)	<0.0001
PVC—*n* (%)	6 (3.2)	5 (3)	1 (4.2)	0.98
PSVC—*n* (%)	7 (3.7)	5 (3)	2 (8.3)	0.32
Simultaneous PVC and PSVC—*n* (%)	2 (1)	2 (1.2)	0 (0)	N.A.
Myopericarditis—*n* (%)	2 (1)	2 (1.2)	0 (0)	N.A.
Others ^§^—*n* (%)	4 (2)	4 (2.4)	0 (0)	N.A.

Abbreviations: AF, atrial fibrillation; PVC, premature ventricular complex; PSVC, premature supraventricular complex; ^§^ includes Takotsubo syndrome (*n* = 1), Brugada-like pattern (*n* = 1), δ wave (*n* = 1), and sinus arrhythmia (*n* = 1).

**Table 4:** Multivariate analysis for 28-day mortality.

Multivariate Adjusted Cox Hazard Regression Model for 28-Day Mortality	Adjusted Hazard Ratio (aHR) *	95% Confidence Interval	*p*-Value
Age (>65 years)	6.38	1.10–37.01	0.039
Male Sex	2.26	0.87–5.88	0.093
D-dimer (>850 U/L)	2.07	0.60–7.12	0.244
AF **	3.02	1.03–8.81	0.042
Right ventricular strain	2.94	1.01–8.55	0.047
QTc interval (>451 ms)	3.24	1.09–9.62	0.033
Tp-e/QTc (>0.20)	0.79	0.28–2.20	0.662

Abbreviations: COPD, chronic obstructive pulmonary disease; AF, atrial fibrillation; QTc, corrected QT interval; Tp-e, T wave peak-end. * adjustment for comorbidities (coronary artery disease, systemic hypertension, chronic heart failure, COPD) and severity of SARS-CoV2 infection (expressed as PaO_2_/FiO_2_ ratio < 300). ** cumulative AF (admission + in-hospital development).

## Data Availability

All data relevant to the study are included in the article and are available from the corresponding author upon request.
